# Restricted Visual Scanpaths During Emotion Recognition in Childhood Social Anxiety Disorder

**DOI:** 10.3389/fpsyt.2021.658171

**Published:** 2021-05-17

**Authors:** Johan Lundin Kleberg, Emilie Bäcklin Löwenberg, Jennifer Y. F. Lau, Eva Serlachius, Jens Högström

**Affiliations:** ^1^Centre for Psychiatry Research, Department of Clinical Neuroscience, Karolinska Institutet, & Stockholm Health Care Services, Region Stockholm, Stockholm, Sweden; ^2^Department of Molecular Medicine and Surgery, Karolinska Institutet, Stockholm, Sweden; ^3^Institute of Psychiatry, Psychology and Neuroscience, King's College London, London, United Kingdom

**Keywords:** social anxiety disorder, eye tracking, visual scanpaths, social attention, child and adolescent, attention bias, emotion

## Abstract

**Background:** Social anxiety disorder (SAD) has its typical onset in childhood and adolescence. Maladaptive processing of social information may contribute to the etiology and maintenance of SAD. During face perception, individuals execute a succession of visual fixations known as a scanpath which facilitates information processing. Atypically long scanpaths have been reported in adults with SAD, but no data exists from pediatric samples. SAD has also been linked to atypical arousal during face perception. Both metrics were examined in one of the largest eye-tracking studies of pediatric SAD to date.

**Methods:** Participants were children and adolescents with SAD (*n* = 61) and healthy controls (*n* = 39) with a mean age of 14 years (range 10–17) who completed an emotion recognition task. The visual scanpath and pupil dilation (an indirect index of arousal) were examined using eye tracking.

**Results:** Scanpaths of youth with SAD were shorter, less distributed, and consisted of a smaller number of fixations than those of healthy controls. These findings were supported by both frequentist and Bayesian statistics. Higher pupil dilation was also observed in the SAD group, but despite a statistically significant group difference, this result was not supported by the Bayesian analysis.

**Conclusions:** The results were contrary to findings from adult studies, but similar to what has been reported in neurodevelopmental conditions associated with social interaction impairments. Restricted scanpaths may disrupt holistic representation of faces known to favor adaptive social understanding.

Social anxiety disorder (SAD) is a highly disabling mental health disorder characterized by intense fear of social evaluation, often leading to extreme distress and avoidance of social interaction (1). SAD has a typical onset in late childhood or adolescence, and if left untreated often takes a chronic course (2). Current theoretical models suggest that etiological factors for SAD include genetic predispositions, temperament, cognitive biases, negative life events, peer-relations and parent behavior, that interact to produce SAD (3). The influential model by Clark and Wells (1995) (4) proposes that when an individual with social anxiety enters a social situation, a set of assumptions and beliefs are activated concerning how they think they have to act and what other people expect of them. A prediction of failure is made which causes the social situation to be perceived as dangerous and to protect the individual from harm, a number of behavioral, cognitive, attentional, affective and somatic processes are activated. These processes, however, are also involved in the long-term maintenance of social anxiety, which prevents habituation to social situations even as the individual engages in frequent interactions with others (5–7). Several models also implicate aberrant information processing in the maintenance or development of SAD (8).

So far, most studies of information processing in SAD have been conducted in adults (9–11). Adults with SAD tend to interpret social signals from others as indications of rejection or hostility (11), a bias that might be driven by disrupted allocation of attention, to socially threatening stimuli (9, 10). Individuals with SAD may also differ from healthy controls regarding the type of stimuli they perceive as threatening. According to the fear of positive evaluation hypothesis, individuals with SAD perceive social cues signaling both negative evaluation (such as an angry face) and positive evaluation (such as a smiling face) as threatening (12, 13).

Since an extensive literature has documented that the development of social attention and emotional brain mechanisms is protracted and often nonlinear, it is not clear whether these results generalize to the younger age range where SAD typically has its onset (14). For example, the period from late childhood to early adulthood is characterized by developmental changes in the relative balance between brain functions involved in automatic emotional reactivity such as the amygdala, and those involved in top-down control of attention and emotion regulation such as the anterior cingulate (ACC) and prefrontal cortex (14–16).

Late childhood and adolescence is a period of high plasticity and intense maturation of the social brain (17, 18). Therefore, aberrant information processing during this period is likely to have cascading developmental consequences (19). Conversely, late childhood and adolescence may be a period when interventions tailored to address disrupted information processing mechanisms are particularly likely to carry long-term benefits (14). To understand these mechanisms is therefore an important research priority.

There is some evidence that youth with SAD misinterpret signs of both negative and positive emotions in faces (20), and show atypical functioning of brain regions involved in face processing (21, 22). However, the patterns of visual attention linked to these observed SAD-linked anomalies are poorly understood.

Visual attention unfolds through a succession of fixations and saccades known as the *scanpath*. Fixated locations are highly prioritized for further cortical processing, and the scanpath is therefore a fundamental aspect of visual information processing. Cognitively demanding tasks typically lead to longer and more widely distributed scanpaths, reflecting a higher degree of mental effort and cognitive control of attention (23, 24). Stimuli signaling potential threats may also be viewed with longer scanpaths than neutral stimuli, possibly driven by increased allocation of attention (25). Some types of facial information can be identified with scanpaths consisting of as little as two fixations, including the identity of familiar individuals or emotional expressions which can be identified by a single feature (e.g., a furrowed eyebrow (26). More typically, novel faces are scanned with multiple fixations (27, 28). A spatially distributed scanpath is believed to facilitate encoding of the face into a holistic percept (26, 29), and improves face recognition (30). Holistic processing is a hallmark of face processing in healthy individuals (31), while individuals with impaired face processing abilities, including those with neurodevelopmental disorders, often use a piecemeal or detail focused strategy (32–34). Restricted scanpaths could be a marker of this detail-focused processing style (35).

So far, a small number of eye-tracking studies have been conducted in children and adolescents with SAD (36–42). These studies examined the relative distribution of attention between threat-related faces and other stimuli during free viewing tasks. Studies using free-viewing tasks are informative about how individuals with SAD spontaneously distribute their attention in the absence of an explicit task and are therefore likely reflecting multiple cognitive processes. However, results from free-viewing tasks are inconclusive, with reports of both avoidance (38, 39), prolonged monitoring of threat (41, 42), quicker orienting to angry faces (43) and equally quick orienting to and from emotional faces in youth with SAD and controls (36). Previous studies are limited by small sample sizes, typically ranging between 20 and 35 individuals [for reviews, see (9, 10)]. Studies in adults have suggested that individuals with SAD look less at the eyes of images of faces when accumulated looking time over several seconds is considered (9). Studies in children and adolescents have so far not reported reduced overall looking time at the eyes in SAD (38, 40). Looking time at the eyes or other regions of a face accumulated over several seconds is likely reflecting several attentional processes, and more temporally sensitive metrics may be needed to detect atypical social attention in child and adolescent SAD (41).

## Scanpaths in SAD

Scanpath measures could provide important information about information processing strategies in SAD but have so far not been examined in pediatric samples. Previous studies in adults with SAD reported a pattern of atypical scanpaths during face perception termed hyperscanning [e.g., (44)]. Hyperscanning can be defined as atypically long and widely distributed scanpaths (42–44). Typically, scanpath length is positively correlated with the number of executed fixations in both healthy individuals and individuals with SAD (25, 44–46). However, one study in adults with SAD reported the opposite pattern – i.e., that individuals who made longer scanpaths also made a smaller number of fixations. One reason for this unusual pattern may be that short fixations with a duration of <200 ms which may be more frequent during hyperscanning were discarded from these analyses (47, 48).

Hyperscanning was initially reported during free viewing of static images of faces (47, 48), and later also for dynamic stimuli during a public speaking task (44). Wermes and colleagues (45) extended these findings and found hyperscanning in adults with SAD but only after an anxiety induction procedure. In contrast, the type of visual stimuli (search for threat or neutral stimulus) did not modulate the results. Finally, Boll and colleagues (49) did not observe a group difference in scanpath length between adults with SAD and healthy controls during an emotion classification task.

## Pupil Dilation in SAD

Theoretical models of SAD propose that enhanced perception of threat during social-evaluative situations affect not only allocation of attention, but also physiological arousal (3). Heightened arousal is a common aspect of anxiety, and social fear could therefore potentially lead to hyperarousal. Consistent with this, brain imaging studies have shown amygdala hyperreactivity during face processing in SAD (21, 22). So far, little is known about potential links between atypical scanpaths and arousal in SAD.

Pupil dilation is an index of arousal directly controlled by joint activity in the sympathetic and parasympathetic branches of the autonomic nervous system. At least two components of the pupil response can be distinguished during stimulus processing. The first is a rapid constriction and subsequent dilation caused by changes in in luminance called the pupillary light reflex (PLR). The second and slower component (the pupil dilation response, PDR) is characterized by a relative increase in pupil size during periods of attention, mental effort, and arousal. This later response is modulated by cholinergic and noradrenergic activity (50, 51). Traditionally, only the PDR has been linked to cognitive processing, but recent studies indicate that also the PLR is affected by such factors (52).

In light of previous studies, it could therefore be expected that SAD would be associated with enhanced pupil dilation to emotional faces. However, this has not been found in the two studies published so far (38, 40). Keil and colleagues examined pupil dilation in 10–13 year old children and controls during face processing (38). Groups did not differ in the amplitude of their pupil dilation response measured during the whole trial interval (10 s), but the SAD group had a larger PLR than controls, which may reflect blunted cognitive modulation of the PLR. A recent study from our group examined the time course of pupil dilation in a group of adolescents with SAD as well as the amplitude of the response (40). Although adolescents with SAD did not differ from healthy controls in pupil dilation amplitude, an atypical time course was found, characterized by a decrease in pupil dilation over the course of stimulus presentation. We sought to extend these results in a larger sample and in the current study we examined pupil dilation amplitude which is the most commonly studied measure in the literature on pupil dilation (53).

## Analysis Plan

The analysis plan was pre-registered in the Open Science Framework after data collection but prior to analysis (link: https://osf.io/dytnf).

## Hypotheses

The following hypotheses were tested:

Hypotheses 1: Youth with SAD will show longer scanpaths than healthy controls during face processing (longer total scanpath and more dispersion between fixations).
Hypothesis 2: Youth with SAD will show a blunted pupil dilation response during later stages of face processing (e.g., after initial adaptation to light).

We did not hypothesize group differences in accumulated looking time to the eyes or mouth but included exploratory analyses of these metrics.

## Methods

### Participants With SAD

Participants aged 10–17 years with SAD were recruited from an ongoing clinical trial evaluating the efficacy of internet-delivered cognitive behavioral therapy (ICBT) for pediatric SAD. Initially, 107 individuals with SAD were invited and 64 of these accepted to participate in the study and completed the experiment. Three participants with SAD were excluded from all analyses because of invalid data (see *Recording and processing of eye tracking data and Statistical Analysis*), resulting in a sample size of 61. A principal diagnosis of SAD according to DSM-5 (1) criteria was confirmed by an experienced clinical psychologist interviewing the child and parents jointly with the Anxiety Disorders Interview Schedule [ADIS; (54)]. The ADIS interview is normally conducted with the child and parents separately, so to ensure that the child's account was given sufficient attention during the interview, parents were instructed to let the child respond first to all questions. Exclusion criteria were initiation or dose modification of psychotropic drug within the past 6 weeks, current psychosis, eating disorder, severe depression, suicidal behavior, or other current severe mental disorder including autism spectrum disorder, or substance or alcohol abuse. Comorbid diagnoses were specific phobia (*n* = 5), generalized anxiety disorder (*n* = 8), depression (*n* = 4), attention deficit/hyperactivity disorder (*n* = 3), separation anxiety (*n* = 1) and panic disorder (*n* = 1). Two individuals with SAD medicated with selective serotonin reuptake inhibitors (SSRIs), three with stimulants (lisdexamphetamine), and one with melatonin. All results remained when participants on medication were excluded.

### Recruitment of the Healthy Control Group

We planned for a control group of *n* = 40. Participants were randomly selected from the Swedish tax registry and contacted by mail. Initially, addresses of 326 10–17 year old children living in the Stockholm area were randomly selected from the Swedish tax registry. Families were sent a letter describing the purpose of the study and were later contacted over telephone and asked to participate. Of the initial sample, 153 families did not respond to the telephone calls, and 107 declined to participate. The remaining 66 participants were asked screening questions before inclusion. Of these, 18 were excluded because of current or previous mental health diagnoses (SAD: *n* = 4, ADHD: *n* = 10, bipolar disorder: *n* = 1, chromosome abnormalities, *n* = 1, obsessive compulsive disorder, *n* = 1, autism, *n* = 1). Seven participants were initially included but did not complete the testing procedure because no suitable time was found. The remaining 41 individuals were included and completed the testing procedure.

Control participants were assessed by a clinical psychologist using the MINI-KID (55). No participant in the control group had a mental health disorder according to the clinical assessment. Two individuals were excluded from analysis because of invalid data, resulting in a sample size of 39. As expected, both youth- and parent reports indicated higher levels of social anxiety in the SAD group than in healthy controls (see Table 1). All participants in the control group scored within one standard deviation of the mean scores on the Liebowitz Social Anxiety Scale – child version (LSAS-C) previously reported in normative samples of youths (56), and could therefore be considered healthy and non-anxious. The LSAS is a self-report measure of fear and avoidance in 24 different social and performance situations, available for youth as well as parents. Groups were matched on age and gender (Table 1). The sample is partly overlapping with the second cohort in a previous study by (41), where data from another experiment are reported.

**Table 1:** Demographics, clinical characteristics and number of valid trials.

	**SAD (*n* = 61)**		**Healthy controls (*n* = 39)**		
	**Mean (SD)**	**Range**	**Mean (SD)**	**Range**	**p**
**Background**					
Age	14.43 (2.19)	10–17.9	14.21 (2.21)	10.30–17.30	0.618
Gender (% Female)	77	–	68		0.355
LSAS (Child report)^1^	79.00 (27.78)	24–135	19.77 (12.25)	2–47	<0.001^***^
LSAS (Parent report)^2^	87.62 (26.06)	34–130	13.63 (12.99)	0–58	<0.001^***^
**Mean nr of valid trials (max possible** **=** **24)**					
Scanpath analysis	21.45 (2.73)	13–24	20.79 (3.42)	13–24	0.311
Pupil dilation	22.92 (1.38)	18–24	22.62 (1.39)	19–24	0.285
**%Correctly identified**					
Angry	98 (4)	88–100	94 (9)	63–100	0.009**
Fearful	95 (10)	63–100	95 (9)	63–100	>0.90
Happy	99 (4)	83–100	97 (7)	75–100	0.203

1 *Based on n = 59 in the SAD group;*

2 *. Based on n = 58 in the SAD group;*

*** *p < 0.001;*

* p < 0.01. SD, standard deviation. LSAS, Liebowitz Social Anxiety Scale.

### Ethical Considerations

The authors assert that all procedures contributing to this work comply with the ethical standards of the relevant national and institutional committees on human experimentation and with the Helsinki Declaration of 1975, as revised in 2008. The study was approved by the Stockholm regional research ethics committee (decision number 2017/1142-31/4).

### Experimental Task

**Figure 1:**
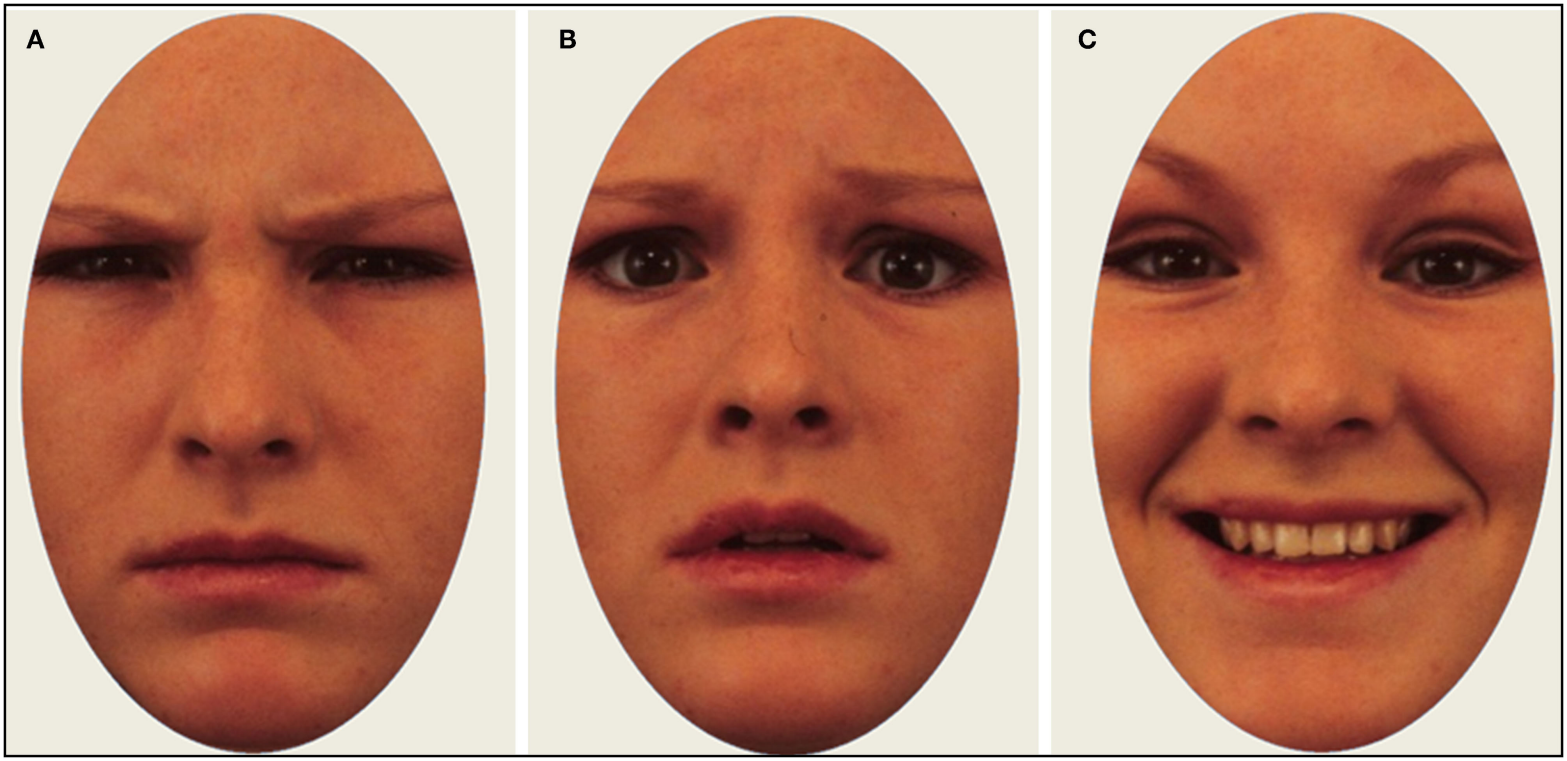
Example of stimulus images with actors displaying an angry **(A)**, fearful **(B)**, and happy **(C)** expression.

Images from a standardized database of actors displaying emotional expressions were used as stimuli (57). In total, 24 images were shown to each participant, evenly distributed between three emotional expressions (angry, happy, fearful)^1^. The same actors appeared once with each expression, meaning that the stimulus set contained eight unique actors (50% male, 50% female). Stimulus images were cropped to show only the inner regions of the face. Each trial began with a fixation cross on a uniform gray screen for 2 s, followed by an emotional face presented for 4 s. Immediately after stimulus offset, participants were asked to identify with a mouse click whether the depicted person felt angry, happy, or fearful. Participants were not asked to make a speeded response. We chose a presentation time of 4 s to give the participants enough time to identify the emotional expressions, and also execute enough eye movements to calculate scanpath metrics. Example of stimulus images are shown in Figure 1.

### Recording and Processing of Eye Tracking Data

Data were recorded using a Tobii X-120 corneal reflection eye tracker (Tobii Inc, Danderyd, Sweden), which samples gaze at 120 Hz and pupil size at 40 Hz. Raw eye tracking data were processed using custom scripts written in MATLAB version 2019a (Mathworks, Inc). Fixations were identified using an I-VT filter (58) with parameters set according to recommendations by the manufacturer. Following linear interpolation of gaps in the data shorter than 100 ms, a moving average filter with a window size of 25 ms was applied to the x- and y-coordinates. Saccades were identified as periods with between-samples velocity exceeding 30°/s, and fixations were defined as periods between saccades. Subsequent fixations within 1° were merged. Fixations shorter than 50 ms were discarded.

**Figure 2:**
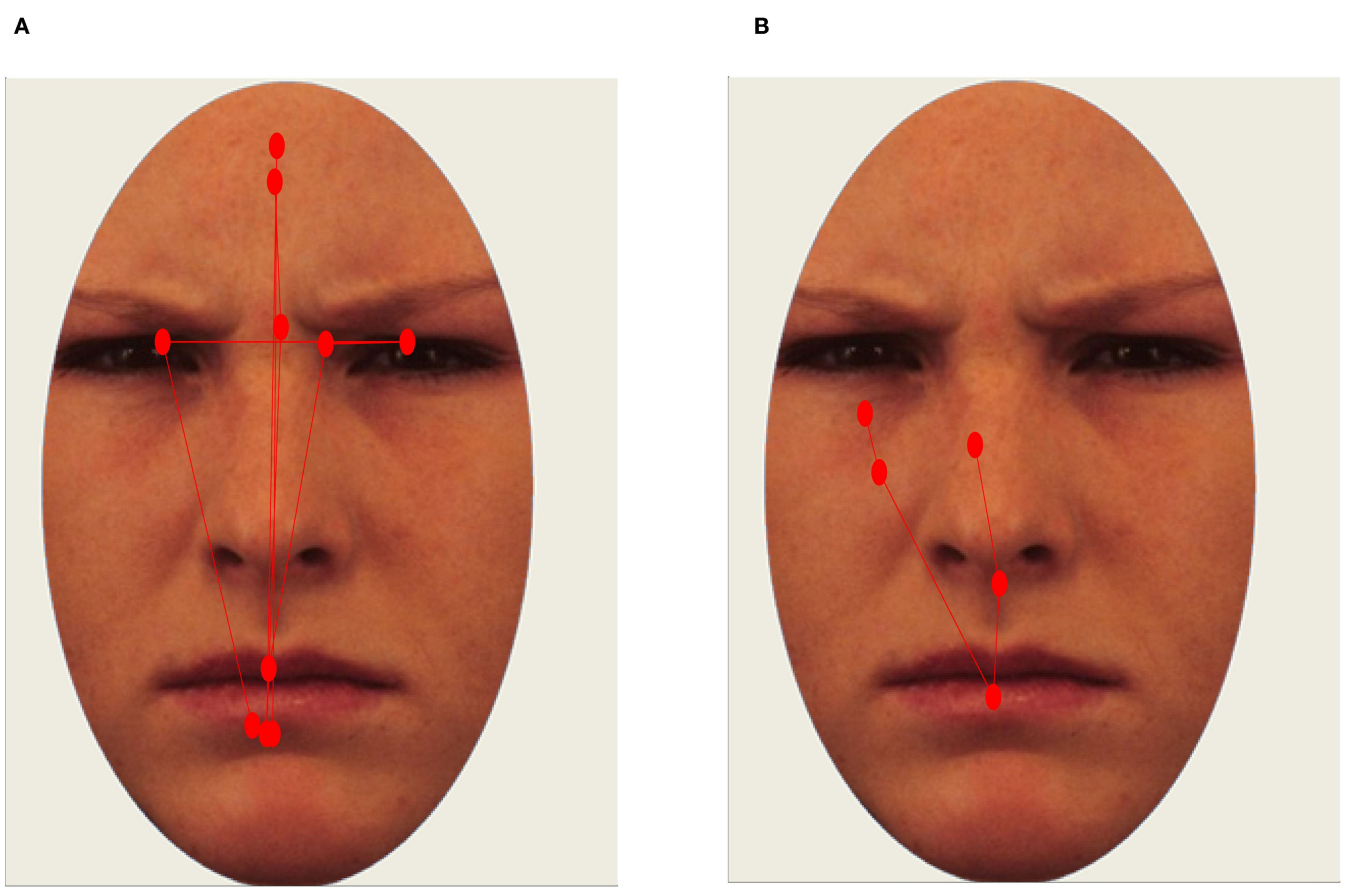
Example of a long scanpath [**(A)**; scanpath length and RMS values above 75th percentile. Healthy participant] and restricted scanpath [**(B)**; scanpath length and RMS values below 25th percentile. Participant with SAD]. Red circles represent fixations.

Two pre-registered scanpath metrics were calculated: (1) The summed Euclidean distance between subsequent fixations (scanpath length); and (2) The root-mean-square (RMS) value of all fixations (scanpath dispersion). The RMS was calculated for each trial by (1) taking the average of the squared deviations from the mean of the x- and y-values of all fixations and (2) taking the square root of these values, and (3) averaging values for the x- and y-coordinates. Higher RMS values therefore reflect a higher degree of spatial dispersion of fixations, whereas scanpath length reflects the total distance that gaze travels during the entire stimulus presentations (see Figure 2 for illustrations). The RMS of individual fixations should not be confused with the RMS of the unfiltered samples constituting a fixation, which is sometimes used as a quality metric in eye-tracking studies.

Scanpath length and dispersion are expressed in degrees of visual field. To account for the fact that more valid data from a trial would also result in longer scanpaths, we divided the scanpath measures by the total valid fixation time (defined as all successfully recorded samples identified as part of a fixation) in seconds. Previous studies of scanpaths in SAD have also analyzed the average number of fixations by trial [fixation count [e.g., 42–44]], a measure typically closely correlated with scanpath length. We included fixation count as an additional metric in the analyses to facilitate comparison with previous studies. Trials with <1,500 ms valid fixation time were discarded.

Pupil data were filtered according to procedures described in an earlier publication from our group (40). Gaps in the data shorter than 150 ms were replaced through linear interpolation and subsequently filtered by a moving median filter corresponding to 80 ms. A pupil dilation response was calculated for each trial and defined as the mean proportional change in pupil size during stimulus presentation relative to baseline pupil size. As in previous publications (40, 59), the initial increase in pupil size after stimulus onset that can largely be attributed to changes in screen luminance was discarded. Based on visual inspection of the data, pupil dilation response was defined as the mean pupil size during the 1,500–4,000 ms interval (see Supplementary Figure 1).

Baseline pupil size was measured during a 750 ms interval directly preceding the stimulus. An estimate of baseline pupil size was calculated for each participant by taking the mean pupil size during this interval from all valid trials. Values outside the +/− 3 SD range from the mean of each participant were considered outliers and were discarded.

### Statistical Analyses

For each individual, a mean of all valid trials within each condition was computed. All data from an individual and condition were rejected if <4 trials were valid. Six participants (3 with SAD) were excluded from all analyses because they were lacking data from all conditions. Cohens' *d* is reported as a standardized effect size of group differences. Age and sex were added as covariates in all analyses. A power analysis indicated that the study had 80% power to detect small to medium effect sizes of *d* = 0.2 or higher.

Hypotheses were tested using generalized linear mixed effects models (GLMM) with random intercept for participant (i.e., treating multiple observations from the same individual as repeated measures). Statistical tests for an effect were performed by comparing a model including the effect to the most complex model without the effect in question (the *null model*). For example: a main effect of group can be tested by comparing the full model Y ~ GROUP + (1|ID) to a null model including only the intercept: *Y* ~1+ (1|ID), where Y is the response variable. Both frequentist statistics (i.e., *p*-values) and Bayesian analyses were conducted. Bayesian statistics have been proposed as an alternative to null hypothesis significance testing (NHST). In a Bayesian analysis, the relative evidence for a hypothesis and the null hypothesis given the observed data can be quantified in terms of a *Bayes factor*. Consequently, researchers may potentially not only conclude that the null hypothesis could not be rejected, but also that it may be supported. Bayesian statistics may be more robust to false positive results than NHST (60). Therefore, we interpreted our results based on the Bayesian statistics when results from the two statistical approaches were conflicting.

In a frequentist framework, the full- and null models were compared using χ^2^-tests, yielding a *p-*value for the significance of the effect (61). The full- and null-models can also be compared using a Bayes factor (BF), expressing the relative likelihood of the two models. Following Wagenmakers (60), a BF is calculated from the Bayesian information criterion (BIC) values of the two models using the following equation:

$${\text{BF}}_{10}={\text{exp}}^{(\text{BIC}\_\text{H}0-\text{BIC}\_\text{H}1)/2}.$$

Where BF_10_ is the Bayes factor favoring H_1_ over H_0_, with higher numbers indicating more evidence supporting H_1_. By reversing the terms, a BF_01_ can be calculated, with higher numbers indicating more support for H^0^. By convention, a BF > 3 indicates positive evidence for the hypothesis, a BF > 20 indicates strong support, and a BF > 150 very strong support (60). *Post-hoc* analyses were conducted to examine whether the observed group differences in scanpath metrics would also be linked to symptom levels of SAD. These analyses were conducted with linear regression models with the mean of all valid trials across conditions as dependent variable. For each participant, the highest value of the child and parent version of the LSAS was used as a measure of SAD symptoms. Sex and age were added as covariates. Bayes factors were computed by comparing a model including SAD symptoms to the next most complex model (the null model). Additional analyses were also conducted to compare accumulated looking time at the eyes and mouth.

## Results

### Preliminary Analyses

No group differences were found in the number of completed trials (see Table 1). As can be seen in Table 1, although correct identification of emotion was close to ceiling in all conditions, the SAD group was more accurate than the HC group in identifying expressions of anger, whereas no group differences were found for happy or fearful faces.

A main effect of emotion was found on scanpath length, so that scanpaths were shorter during processing of happy compared to fearful and angry expressions. No effects of emotion were found on scanpath dispersion. Expressions of anger and fear elicited higher pupil dilation than expressions of happiness. These results are shown in Table 2. Groups did not differ in overall looking time at the eyes (χ^2^ = 0.12, *p* = 0.730, BF_10_ = 0.11, *d* = 0.07) or mouth (χ^2^ = 0.32, *p* = 0.575, BF_10_ = 0.12, *d* = 0.09). There were also no interactions between group and emotion in looking time at either region (see Supplementary Table 1).

**Table 2:** Main effects of emotion for the studied dependent variables.

	**χ^2^**	**p**	**b**	**SE**	**BF_**10**_**	**BF_**01**_**	**d**
**Scanpath Length**							
Fearful > Angry	1.33	0.249	0.17	0.15	0.20	**5.16**	0.08
Angry > Happy	6.26	0.012^*^	−0.34	0.14	2.30	0.44	0.15
Fearful > Happy	14.08	<0.001^***^	0.51	0.13	**114.91**	0.01	0.22
**Scanpath Dispersion**							
Fearful > Angry	1.04	0.307	−0.02	0.02	0.17	**5.91**	0.06
Angry > Happy	1.18	0.277	−0.03	0.02	0.18	**5.52**	0.07
Fearful > Happy	0.03	0.859	0.00	0.02	0.10	**9.80**	0.02
**Pupil Dilation**							
Fearful > Angry	4.85	0.028^*^	−0.57	0.25	1.12	0.90	0.28
Angry > Happy	18.32	<0.001^***^	−1.22	0.27	**>500**	<0.01	0.57
Fearful > Happy	5.92	0.015^*^	0.65	0.26	1.89	0.52	0.29

*** *p < 0.001;*

* *p < 0.05.*

*b, unstandardized beta coefficient; SE, standard error; BF_10_, Bayes factor favoring the alternative hypothesis; BF_10_, Bayes factor favoring the null hypothesis; d, Cohen's d*.

### Main Analysis (Registered Hypotheses)

#### Scanpath Length

**Figure 3:**
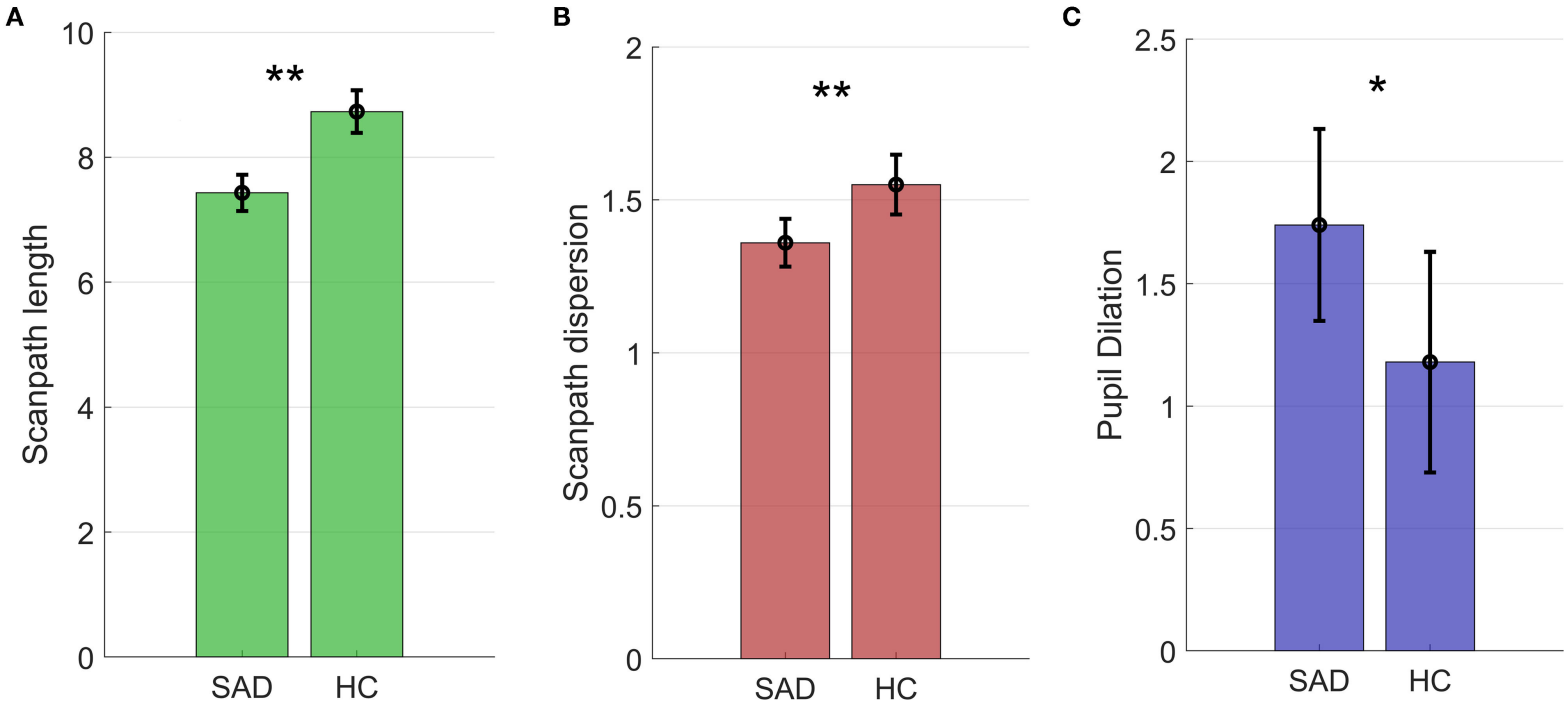
Scanpath length in degrees of the visual field **(A)**, scanpath dispersion **(B)**, and pupil dilation **(C)** in the SAD and HC groups. Figures show estimated marginal means and 95% confidence intervals *p < 0.01; **p < 0.05.

Results are shown in Table 3 and Figure 3 and summarized here. Scanpaths were shorter in the SAD group than in healthy controls (χ^2^ = 9.38, *p* = 0.002, BF_10_ = 10.94, *d* = 0.54). This effect was not qualified by any interaction effect between group and emotion (χ^2^ = 3.60, *p* = 0.167, BF_10_ = 0.06).

**Table 3:** Group differences between youth with SAD and healthy controls in scanpath length, scanpath dispersion and pupil dilation response.

	**M (SD)**		**Group comparison**					
**Measure**	**SAD**	**HC**	**χ^2^**	**p**	**BF_**10**_**	**BF_**01**_**	**d**	**Direction**
Scanpath length (° of visual field)	7.59 (2.02)	8.82 (2.44)	9.38	0.002^**^	**10.94**	0.09	0.54	SAD < HC
Scanpath dispersion	1.36 (0.32)	1.56 (0.42)	7.68	0.006^**^	**4.68**	0.21	0.51	SAD < HC
Fixation count	9.17 (1.53)	10.14 (1.50)	15.31	<0.001***	**211.90**	0.01	0.62	SAD < HC
Pupil dilation response	1.13 (2.08)	1.75 (2.18)	4.58	0.032^*^	0.98	1.03	0.29	SAD > HC

** *p < 0.01;*

* *p < 0.05. b, unstandardized beta coefficient; SE, standard error; BF_10_, Bayes factor favoring the alternative hypothesis; BF_10_, Bayes factor favoring the null hypothesis; d, Cohen's d; HC, Healthy Control*.

#### Scanpath Dispersion

Scanpaths were also less dispersed in the SAD group compared to healthy controls (χ^2^ = 7.68, *p* = 0.006, BF_10_ = 4.68, *d* = 0.51; see Table 3 and Figure 2). Again, no interaction between group and emotion was found (χ^2^ = 0.43, *p* = 0.806; BF_10_ = 0.01). To sum up, restricted scanpaths were observed in the SAD group, a conclusion supported by both Bayesian and frequentist statistics.

#### Fixation Count

As can be seen in Table 3, participants in the SAD group made a smaller number of fixations than healthy controls (χ^2^ = 15.31, *p* < 0.001, BF_10_ = 211.90, *d* = 0.62). No interaction between group and emotion was found (χ^2^ = 0.27, *p* = 0.88, BF_10_ = 0.01).

#### Relation Between Scanpaths and Symptoms of SAD

We conducted *post-hoc* analyses to examine whether symptom levels of SAD were linked to scanpath metrics. Higher levels of SAD symptoms were linked to shorter scanpath length (β = 0.24, *p* = 0.015, BF_10_ = 2.29) and smaller number of fixations (β = 0.32, *p* = 0.001, BF_10_ = 24.70). No relation was found between SAD symptoms and scanpath dispersion (β = 0.04, *p* = 0.32, BF_10_ = 0.17).

#### Pupil Dilation

The SAD group had higher pupil dilation than controls. The difference was statistically significant (χ^2^ = 4.58, *p* = 0.032, *d* = 0.29), but the Bayes factor indicated that the data were marginally more likely under the null hypothesis (BF_10_ = 0.98, equivalent to BF_01_ = 1.03), and that the data were therefore inconclusive (see Figure 3 and Table 3). No interaction between group and emotion was found (χ^2^ = 0.05, *p* = 0.792, BF_10_ = 0.01).

#### Relation Between Pupil Dilation and Scanpaths

Exploratory *post-hoc* analyses were conducted to examine links between pupil dilation and scanpath measures. No relations were found between pupil dilation and scanpath length (β = −0.11, *p* = 0.22, BF_10_ = 0.22, BF_01_ = 4.59), between pupil dilation and scanpath dispersion (β = −0.15, *p* = 0.10, BF_10_ = 0.40, BF_01_ 2.50), or pupil dilation and number of fixations (β = 0.04, *p* = 0.69, BF_10_ = 0.40, BF_01_ = 9.29). The Bayes factors favored the null hypothesis.

## Discussion

Social anxiety disorder in children and adolescents is associated with cognitive biases that may maintain or exacerbate symptoms. One of the factors underlying these biases may be a pattern of disrupted allocation of attention during information processing. The current study examined visual scanpaths and pupil dilation during emotion recognition in children and adolescents with social anxiety disorder. Emotional faces are a social evaluative cue and are as such a disorder relevant stimulus in SAD. Compared to healthy controls, youth with SAD had shorter and less dispersed scanpaths, a finding supported by both frequentist and Bayesian statistics. Further analyses showed that higher levels of SAD symptoms were linked to shorter scanpaths and a smaller number of fixations, again suggesting restricted visual scanning in children with SAD.

These results were contrary to our registered hypothesis, and also contrary to what has been reported in adult studies (44, 47, 48) where it has been proposed that socially anxious individuals scan faces with prolonged scanpaths (10, 44). Our results suggest that this attention pattern is not present in pediatric populations. Instead, a pattern of restricted scanpaths was found. As noted in the introduction, under normal attentional circumstances, wider scanpaths are observed during periods of controlled attention and mental effort. The observed pattern of restricted scanpaths in the SAD group could therefore reflect difficulties with cognitive control and allocation of attention.

Restricted scanpaths may lead to a face processing strategy based on attention to single features rather than global configurations (i.e., holistic face processing). Importantly, the observed pattern of restricted scanpaths in SAD was not modulated by the emotional expression of the facial images. In fact, longer scanpaths to negative emotions (anger and fear) compared to positive (happiness) were observed in both groups, replicating previous findings in healthy populations (25, 62). It is possible that smiling faces with direct gaze may be interpreted as threatening by individuals with SAD, since they signal possible social evaluation (12, 13, 63).

Holistic as compared to detail-focused processing is a hallmark of normal face perception. However, a piecemeal strategy can sometimes facilitate detection of negative emotional expressions such as anger or sadness (64), which can be detected based on single features (65). In the current study, patients with SAD were more accurate in identifying expressions of anger than controls but did not differ in accuracy for happiness or fear. Although this finding should be interpreted with caution due to potential ceiling effects, it suggests that youth with SAD may show superior detection of angry facial affect.

The relation between restricted scanpaths and social anxiety may be bidirectional. Previous studies in nonclinical samples have demonstrated that negative mood is associated with disrupted holistic face processing (64, 66). Similarly, social anxiety may therefore disrupt holistic face processing. It is also possible that disrupted holistic processing exacerbates or maintains social anxiety to the extent that it reduces the ability to interpret ambiguous or complex facial information.

The observed pattern of restricted scanpaths may also reflect patients with SAD needing less information than healthy individuals to identify facial expressions as emotional. Longer scanpaths are associated with task difficulty and complexity (24, 62). The fact that longer scanpaths were observed in the control group could therefore reflect task difficulty. In support of this interpretation, Melfsen and Florin (20) reported that children with SAD had lower perceptual thresholds for identifying facial expressions as emotional. Interestingly, restricted scanpaths to stimuli with social content have previously been reported in other conditions with known social interaction impairments, including autism (35), schizophrenia (32) and schizotypy (29), suggesting that it may be a transdiagnostic mechanism. Atypical scanpaths were found in children with SAD despite the fact that they did not differ in overall looking time at the eyes or mouth.

Our finding of restricted scanpaths in the SAD group are opposite to what has been described in the adult studies (44, 48), pointing to a possible developmental difference between children and adults with SAD. It is possible that restricted scanning may have negative developmental consequences during this period, by restricting opportunities for learning. An interesting question for longitudinal studies is therefore whether restricted scanpaths predict worse social functioning or higher levels of SAD symptoms at later time points.

On the other hand, the transitional phase of adolescence may involve greater plasticity at the behavioral and neural level (14), thus interventions designed to alter these processing anomalies may yield greater and longer-lasting benefits. Interventions targeting SAD symptoms through attention training have been attempted, but evidence for their efficacy is so far limited (14). If these interventions are to be successful, a better understanding of the patterns of attention associated with SAD is needed. Our results suggest that restricted scanpaths may be a feasible target for interventions.

Our second aim was to examine pupil dilation during face processing. The SAD group had higher pupil dilation responses than healthy controls while viewing faces, regardless of their emotional expression. According to a conventional frequentist statistical analysis (i.e., inferential statistics based on *p*-values), this effect was statistically significant. However, the Bayesian analysis indicated that the hypothesis was marginally less likely than the null, rendering the result inconclusive. Bayesian statistics may be less vulnerable to false positives than frequentist statistics (60), and we believe that this result is therefore best interpreted as inconclusive. This means that we were not able to replicate previously reported findings of blunted pupillary reactivity during face perception in pediatric SAD, although it should be noted that these studies examined partly different pupil dilation metrics (38, 40). There was no relation between pupil dilation and any of the examined scanpath metrics, indicating no direct link between reduced scanpaths and hyperarousal.

Some limitations should be noted. Although the present study is one of the largest eye tracking studies of pediatric SAD to date, we were not able to compare individuals with SAD to groups with other mental health disorders. Future studies would also benefit from direct comparisons between child and adult populations with SAD. The generalizability of the findings may also be limited to treatment-seeking individuals with SAD, rather than to a broader population of youths with SAD. It should also be noted that the study did not include non-facial control stimuli. Therefore, it is not clear whether the findings are specific for faces or reflect a more general form of atypical attention. Future studies could benefit from the inclusion of additional experimental conditions, including nonsocial stimuli and more ambiguous and complex facial expressions. Finally, due to the limited sample rate of the equipment (40 Hz), we were not able to examine metrics which are sensitive to timing such as the time course of the pupil dilation response or the PLR amplitude, as was done in previous studies (38, 40). An interesting question for future studies is to examine whether scanpath lengths in children with SAD is related to other types of attention and perceptual judgement, including memory for faces. In a previous adult study (45), hyperscanning was found only after an anxiety induction procedure. An interesting question for future studies is whether scanpaths in youth with SAD are also affected by induction of state anxiety. Studies manipulating gaze behaviors (for example by instructing participants to scan either narrowly or broadly) could also examine whether scanpath length causally affects arousal.

Strengths of the study includes the use of a clinically well-characterized sample of treatment-seeking patients with SAD and a matched control group randomly selected from the general population.

## Data Availability Statement

The raw data supporting the conclusions of this article will be made available by the authors, without undue reservation.

## Ethics Statement

The studies involving human participants were reviewed and approved by Etikprövningsmyndigheten (Swedish Ethical Review Authority). Written informed consent to participate in this study was provided by the participants' legal guardian/next of kin and by participants aged 15 - 17. Participants aged 10 - 14 gave verbal ascent.

## Author Contributions

JK and JH designed the study. JK analyzed the data and drafted the manuscript. JL, EL, and ES contributed to the interpretation of the data. All authors contributed to the article and approved the submitted version.

## Conflict of Interest

The authors declare that the research was conducted in the absence of any commercial or financial relationships that could be construed as a potential conflict of interest.

## Footnotes

^1^The following images from the KDEF database were used as stimuli: ANSF07, ANSF09, ANSF33, ANSM07, ANSM08, ANSM09, ANSM32, HASF07, HASF09, HASF33, HASM07, HASM08, HASM08, HASM32, FESF07, FESF09, FESF33, FESM07, FESM08, FESM09, FESM32.
