# Supplementary material for: Restricted Visual Scanpaths During Emotion Recognition in Childhood Social Anxiety Disorder
Source: Front Psychiatry. 2021 May 17;12:658171. doi: 10.3389/fpsyt.2021.658171 (PMC8165204; doi:10.3389/fpsyt.2021.658171)
Supplement: Supplementary file 1 [file Data_Sheet_1.docx]

Supplementary Materials

**A. Definition of the pupil dilation response**

*
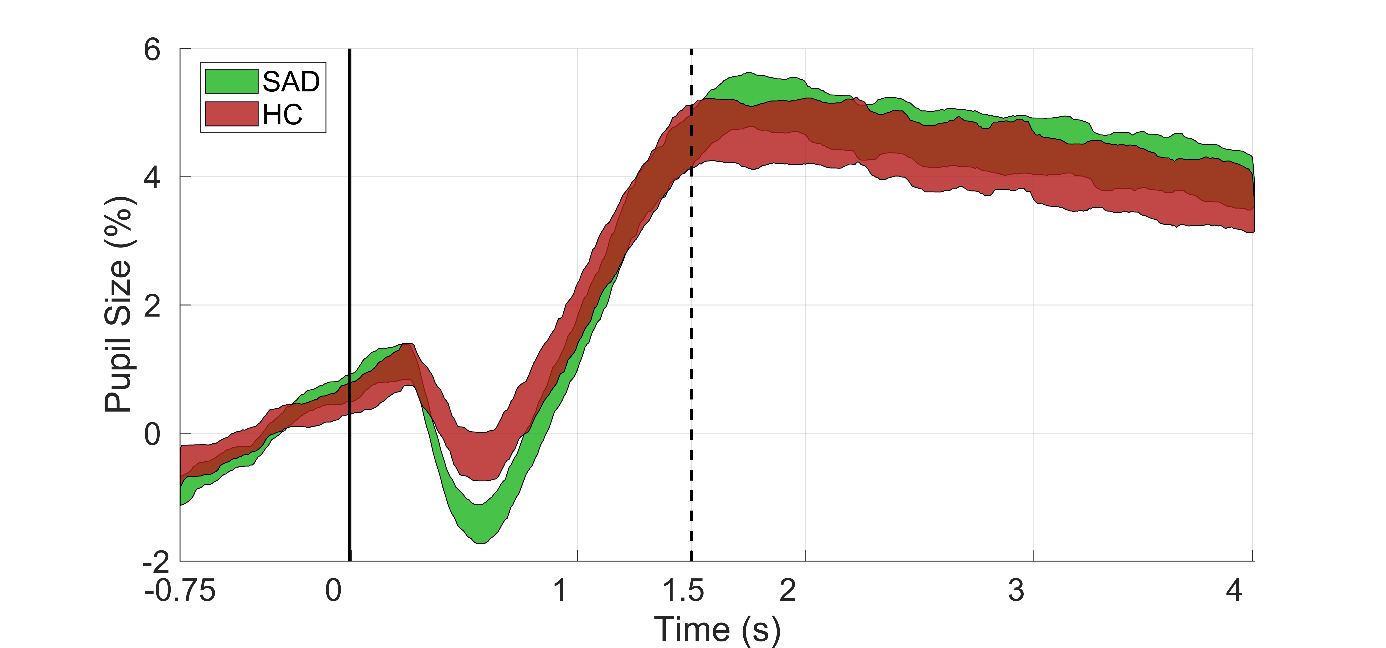
*

**Figure S1***.* Pupil trace as a function of time after stimulus onset in the SAD and HC group (across emotions). Colored areas cover means of all samples +/- 95% confidence intervals. The x-axis shows time relative to stimulus onset. The pupil dilation response was defined as the average pupil size during the 1.5-4 seconds interval, normalized to baseline pupil size. Baseline pupil size was defined as the average pupil size during the 0.75 seconds interval directly preceding the stimulus. A fixation cross was shown during this time-period.

**B. Accumulated looking time at the eyes and mouth**

*
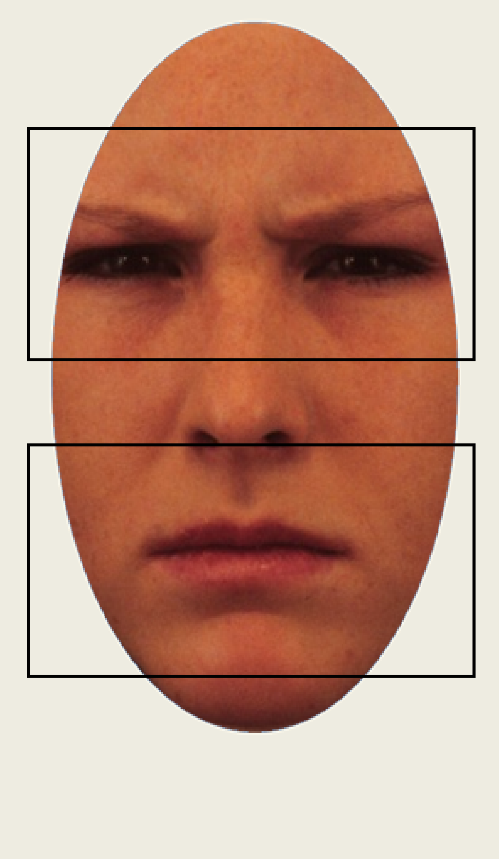
*

*Figure S2. Areas of interest (AOIs) around the eyes and mouth used in the looking time analyses. The AOI:s extend approximately 0.75 degrees of the visual field in each direction from the border of the eyes and mouth, thereby accounting for imprecision in the data.*

*Table S1.* Effects of group (SAD, HC) and emotion (fear, anger, happiness) on looking time at the eyes and mouth.

| **Eyes** | **χ2** | **P** | **b** | **SE** | **BF_10_** | **d** |
| --- | --- | --- | --- | --- | --- | --- |
| **Effects of group** |  |  |  |  |  |  |
| Group (HC > SAD) | 0.12 | .730 | 0.01 | 0.03 | 0.11 | 0.07 |
| Emotion x Group | 0.05 | .977 | 0.01 | 0.02 | 0.02 |  |
| **Effects of Emotion** |  |  |  |  |  |  |
| Fear > Anger | 1.53 | .217 | 0.01 | 0.01 | 0.22 | 0.02 |
| Anger > Happiness | 16.83 | **<.001** | 0.05 | 0.01 | 476 | 0.29 |
| Fear > Happiness | 25.06 | **<.001** | 0.05 | 0.01 | > 500 | 0.33 |
|  |  |  |  |  |  |  |
| **Mouth** |  |  |  |  |  |  |
| **Effects of group** |  |  |  |  |  |  |
| Group (SAD > HC) | 0.32 | .575 | 0.01 | 0.02 | 0.12 | 0.09 |
| Emotion x Group | 2.41 | .299 | 0.02 | 0.02 | 0.03 |  |
| **Effects of Emotion** |  |  |  |  |  |  |
| Fear > Anger | 2.30 | .129 | 0.01 | 0.01 | 0.33 | 0.06 |
| Happiness > Anger | 24.79 | **<.001** | 0.05 | 0.01 | > 500 | 0.40 |
| Happiness > Fear | 34.18 | **<.001** | 0.06 | 0.01 | > 500 | 0.43 |
